# Non-Intrusive Load Monitoring of Buildings Using Spectral Clustering

**DOI:** 10.3390/s22114036

**Published:** 2022-05-26

**Authors:** Muzzamil Ghaffar, Shakil R. Sheikh, Noman Naseer, Zia Mohy Ud Din, Hafiz Zia Ur Rehman, Muhammad Naved

**Affiliations:** Department of Mechatronics and Biomedical Engineering, Air University, Islamabad 44000, Pakistan; muzzamil.ghaffar@mail.au.edu.pk (M.G.); noman.naseer@mail.au.edu.pk (N.N.); drzia@mail.au.edu.pk (Z.M.U.D.); hafizzia@mail.au.edu.pk (H.Z.U.R.); mnaved@mail.au.edu.pk (M.N.)

**Keywords:** non-intrusive load monitoring, energy disaggregation, spectral clustering, graph signal processing, demand-side energy management, smart buildings

## Abstract

With widely deployed smart meters, non-intrusive energy measurements have become feasible, which may benefit people by furnishing a better understanding of appliance-level energy consumption. This work is a step forward in using graph signal processing for non-intrusive load monitoring (NILM) by proposing two novel techniques: the spectral cluster mean (SC-M) and spectral cluster eigenvector (SC-EV) methods. These methods use spectral clustering for extracting individual appliance energy usage from the aggregate energy profile of the building. After clustering the data, different strategies are employed to identify each cluster and thus the state of each device. The SC-M method identifies the cluster by comparing its mean with the devices’ pre-defined profiles. The SC-EV method employs an eigenvector resultant to locate the event and then recognize the device using its profile. An ideal dataset and a real-world REFIT dataset are used to test the performance of these two techniques. The f-measure score and disaggregation accuracy of the proposed techniques demonstrate that these two techniques are competitive and viable, with advantages of low complexity, high accuracy, no training data requirement, and fast processing time. Therefore, the proposed techniques are suitable candidates for NILM.

## 1. Introduction

Energy security is one of the critical factors for the sustainability and integrity of society [1]. The balance between energy supply and demand is vital for energy security. To achieve this balance, monitoring, accounting, and management of energy consumption on the demand side is necessary [2]. Non-intrusive load monitoring (NILM) has been established as a good substitute for intrusive submetering [3], thus becoming a future tool for energy monitoring.

**Figure 1:**
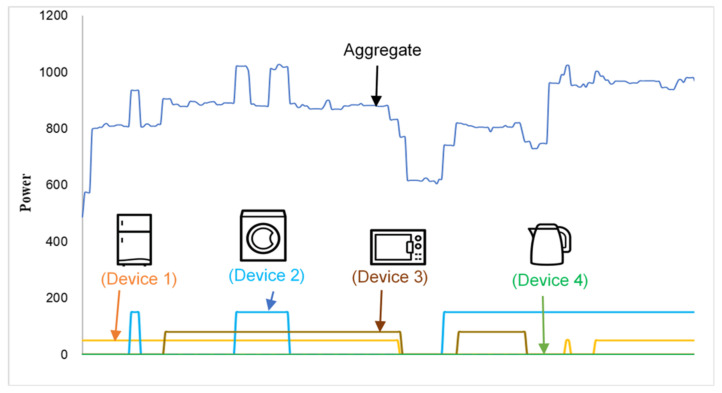
Concept of aggregate load profile and NILM. The task of NILM is to disaggregate the energy of each device and then identify it as shown in the colored plots.

Two critical applications of NILM are home energy management systems (HEMS) and ambient assisted living (AAL) [4], where it provides various solutions to the existing problems and opens avenues for future research. The energy profile of each device is of extreme importance in the overall operation of smart grids with renewable energy resources. NILM plays a vital role in efficiently extracting energy consumption data down to the appliance level, as demonstrated in Figure 1, helping demand prediction. This energy-demand information may be used to manage and conserve energy at the consumer and grid levels.

### 1.1. Motivation

Shortage of energy is a challenge of the current time. An increase in energy production requires investment and dealing with many constraints. Nevertheless, there is much room for improving efficient energy utilization while minimizing the overall losses. NILM forms a basis for efficient energy utilization. NILM research is focused on disaggregating the energy usage of individual appliances attached to a central energy meter. This research area comes under the umbrella of cyber-physical systems and Industry 4.0 [5]. The objective of NILM is to save on the hardware while providing sufficiently accurate energy usage patterns. The rise of NILM is linked with other recent areas of research such as the Internet of Things (IoT) for buildings [6], smart grids, and demand-response management systems [7], where the information from NILM is further utilized for buildings’ energy management and decision making. The primary purpose of all these areas is to manage and conserve energy by enabling stakeholders to make informed decisions.

**Figure 2:**
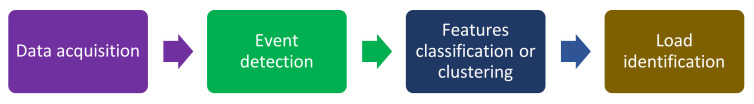
Steps of the NILM process.

The whole process of NILM can be divided into four significant steps, as illustrated in Figure 2. The first step is to acquire the load signature (LS) using a suitable physical sensor. Aggregate real power consumption is taken as LS in this paper. This aggregate real power consumption in buildings is acquired using a commercial smart digital energy meter. The current study is an event-based approach and thus the second step detects individual events. An event is defined as a significant change or perturbation in the aggregate power. Each event is thus considered as corresponding to a state change of a device. After event detection, an important task is to identify or classify the event and the respective device with the help of specific features of the LS, including statistical quantities such as the data mean, peak, slope, median, mode, percentiles, range, variance, and standard deviation. Supervised (classification) and unsupervised (clustering) approaches are used at this point. Repeating this process for each data sample leads to a complete load identification or disaggregation at the individual device level.

Hart [8] initially coined the concept of disaggregating the total energy and demonstrated that each appliance or device could be recognized using an appropriate LS feature, as shown in Figure 1. He also defined the following three types of device models:

- Type 1: ON/OFF;
- Type 2: Finite state machine (FSM);
- Type 3: Continuously variable.

Machine learning approaches can be divided into supervised and unsupervised methods. A labeled dataset is employed in supervised algorithms, which train the algorithm to classify the test data and allocate them to a suitable class, while in unsupervised techniques no labeled training data are required. Although this paper proposes unsupervised methods, a brief overview of related supervised methods is presented below for comparison.

A lot of research has been done in event-based and non-event-based techniques. The research includes both supervised and unsupervised machine learning methods. However, the unsupervised and semi-supervised methods are relatively less explored [9,10]. Unsupervised methods have the advantage of less or no labeled training data required for classification; however, a disadvantage is that they have relatively low classification accuracy.

Some supervised learning approaches that improve accuracy and reduce computational time include artificial neural networks [11,12]. Neural networks are one of the crucial techniques in energy disaggregation. A variant of neural networks are known as concatenated convolutional neural networks (CNNs) [13]: CNN-based algorithms achieve good generalization and energy disaggregation even with a short sample time. Deep neural networks have also been applied to energy disaggregation and give promising results [14,15]: increasing the accuracy of the specific scenario. Another variant called auto-associative neural networks was used on transient-based LS. It was implemented on REDD and UK-DALE datasets [16]. Another approach studied the energy transients using artificial neural networks, improving the accuracy and reducing the computational complexity [17].

Support vector machines [18,19], k-nearest neighbors [20,21], naïve Bayes classifiers [22,23], and linear-chain conditional random fields [24] are also well-known methods. Probabilistic approaches for NILM have also been studied. In one such versatile study, authors explored the Viterbi algorithm with sparse transition and the Markov chain, showing improved performance compared with Bayesian classifiers. In [25], the noise was used as the LS to detect eight appliances. Wavelet transform has also been applied to the transient signature for NILM [26]. In [27], short-time Fourier transform was used to identify different types of devices from transient power, where the shape of the transient data was used for identification. A novel LS called frequency invariant transformation of periodic signals was employed in a steady-state approach. The idea was to use the original electric current waveform with respect to the reference voltage as a signature for NILM. A neural network was employed and an accuracy of 90% was achieved with 18 different devices [28]. In [29], particle swarm optimization was used to optimize the training parameters for the neural network. One of the aspects in NILM research is a reduction in computational complexity. In this direction, authors from [30,31] have adopted a lightweight approach that can run on the edge. A combination of CNN and k-NN was employed to achieve good results.

Unsupervised approaches do not require huge labeled datasets for training [32,33]; instead, they treat the electrical system as a stochastic system and work with unlabeled data. Although unsupervised algorithms are less precise and computationally complex, they can disaggregate devices without training or labeled data. This characteristic can be used in available systems and, on a commercial scale, in ready-to-use NILM systems. One of the unsupervised techniques used for NILM is the hidden Markov model (HMM) and its variants [34,35]. In [35], some variants of the factorial hidden Markov model (FHMM), factorial hidden semi-Markov model (FHSMM), and conditional FHMM (CFHMM) were proposed. HMM is a probabilistic technique with a random model. It assumes that the system has some unobservable states. In [36], an additive and difference FHMM was introduced. A similar but separate study [37] achieved an accuracy higher than 90% for a specific scenario. Here, f-measure (F1) and normalized disaggregation error (NDE) parameters were compared and discussed, showing improved efficiency. The study used a combination of features, i.e., real power, reactive power, and voltage waveforms for five appliances. In [38], a combination of difference HMM and extended Viterbi algorithms was tested. Normalized error and root mean square error were used as performance metrics to compare two variants of this approach. These techniques are generally helpful. Mainly, HMMs perform well in the case of constantly ON devices like the fridge and freezer. However, they are computationally complex and long processing time makes them inappropriate for real-time implementation without increasing processing cost appreciably.

In studies [39,40], six categories of the sampling rate are established:

- Very low: slower than one sample per min;
- Low: between one sample per min to 1 Hz;
- Medium: faster than 1 Hz up to fundamental frequency (fundamental frequency: lowest frequency in the signal);
- High: from the fundamental frequency up to 2 kHz;
- Very high: between 2 and 40 kHz; and
- Extremely high: faster than 40 kHz.

This paper uses the low sampling rate data.

### 1.2. Graph Signal Processing

A relatively new semi-supervised classification technique for NILM, based on graph signal processing (GSP), has been presented in [41,42]. GSP is a field of study that deals with irregular data in time and space, such as a random network of sensors, the internet, and social networks. Data points in the graph are represented as nodes, also called *vertices* (singular vertex). A vertex is one of the points/nodes on which a graph is defined. The vertices are connected through edges. In the graph, the edges represent relationships or interconnections between vertices. The *edges* may be directed or undirected and can even have weights associated with them.

**Figure 3:**
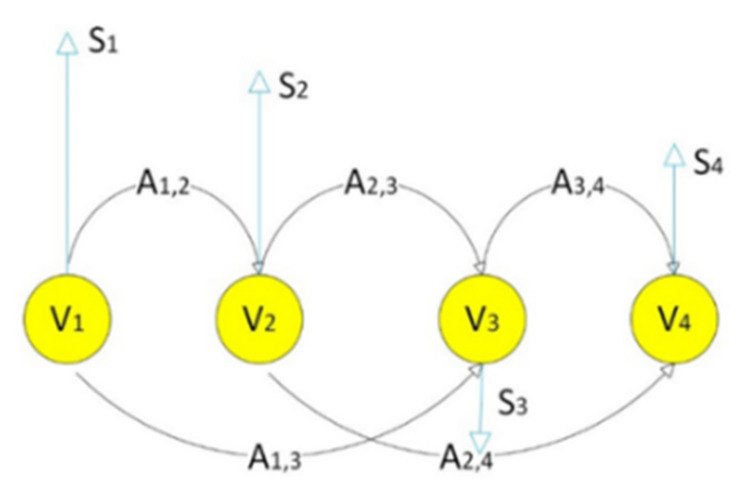
Structure of a graph showing vertices (V_1_ to V_4_), edges *A_i, j_*, and signals (S_1_ to S_4_).

Figure 3 shows a visual representation of the graph with four vertices at data points; these vertices are connected using *edges*. The mathematical model of the graph is $G=\left(V,A\right)$, where each *vertex* 𝓋*_i_* ϵ V and *edges* are represented using the adjacency matrix *A*.

(1) $$A_{i,j}=exp\left\{-\frac{{\left(x_{i}-x_{j}\right)}^{2}}{\sigma^{2}}\right\}$$

where $x_{i}$, $x_{j}$ are two consecutive data samples and σ is a scaling factor. The number of edges connected to the node represents the degree of that node. The degree of each node is obtained using the degree matrix, $D_{j,j}$; it is obtained by adding rows of the adjacency matrix.

(2) $$D_{i,i}=\overset{N}{\underset{j=1}{\sum }}A_{i,j}$$

In Equation (2), *D* is an *N × N* diagonal matrix [42]. Another representation is the Laplacian, which has excellent properties and is very useful in spectral clustering. The Laplacian is mathematically represented as:

(3) L=D−A

Graph signal processing (GSP) is a relatively new and valuable unsupervised technique used for NILM, having many advantages, as discussed earlier. One comprehensive and detailed study by Stankovic [41] explored the application of GSP and proved its applicability. NILM accuracy was further improved in a later study [43] using additional preprocessing and post-processing steps. In another study, the post-processing techniques were further enhanced using optimization and a genetic algorithm (GA) [44]. In one recent study [45], authors adopted GSP and the concept of clustering and produced favorable results and improved computation time. The current work is inspired by these studies and tries to explore GSP further and seek improvement from the NILM point of view.

### 1.3. Spectral Clustering

Less research has been conducted in the unsupervised domain as compared to the supervised domain [46]; particularly, the use of spectral clustering is rarely explored for NILM applications and has room for further exploration and research. This study examines the feasibility of applying spectral clustering in simple yet efficient manner in order to enhance its performance in NILM applications.

Spectral clustering [47,48,49] finds its roots in graph theory; its ultimate task is to cluster out the data based on their edges’ connectivity. This method also allows dealing with non-graphical data. Spectral clustering classifies the data using the eigenvalues (spectrum) of the Laplacian matrix. The concepts of eigenvalues and eigenvectors are of extreme importance here. For a matrix *A*, if there exists a vector *x* that is not all zeros and a scalar λ such that:

(4) Ax=λx

Then x is said to be an eigenvector of *A* with corresponding eigenvalue λ. By careful examination of eigenvalues, it is found that there are some eigenvalues equal to or near to zero, which represent connected components within the graph. The corresponding eigenvectors are constant. The first non-zero eigenvalue is called the spectral gap. The spectral gap gives an approximate idea about the sparsity of the graph. The second eigenvalue is called the Fiedler value, and the corresponding vector is the Fiedler vector. The Fiedler value approximates the minimum graph cut needed to separate the graph into two connected components. However, the number of eigenvalues and vectors depends on the specified upper limit of clusters.

In one recent study [50], automated spectral clustering is applied on multiscale data. The approach used is iterative and obviates the need of predefining parameters. It was tested for NILM applications. In the current proposed study, although various parameters are predefined the approach is simplified and less computationally complex. In another preliminary study [51], spectral clustering has been used for NILM applications. However, the approaches proposed in the current study are different and novel. Moreover, a comprehensive and detailed analysis centered on NILM is presented in the current study.

### 1.4. Current Study

The current study uses graph signal processing in combination with spectral clustering to disaggregate energy data. This study targets the low-frequency range, i.e., between one sample per min to 1 Hz sampling rate from a sampling point of view. Current commercially available smart meters generally provide data in this range. The novel contributions presented in this study are summarized below:

1. This study puts forward two different and novel algorithms based on the spectral clustering classification method, along with a detailed analysis.
  - The first algorithm, designated as the spectral clustering mean (SC-M) method, uses the cluster’s mean to identify the appliance.
  - The second algorithm, designated as the spectral clustering eigenvector (SC-EV) method, uses the spectrum to identify the event and thus disaggregate data.
2. The SC-EV technique proposes a novel idea of determining events based on the sum of the eigenvectors of each cluster. The change of the resultant eigenvector corresponds to an event within a specific threshold limit.

## 2. Methods

For the sake of problem formulation, a set *M* of m appliances is considered *M* = [*D*_1_*,…, D_i_,…, D_M_*], where *D_i_* is the *i*th device. The aggregate active power of the whole house is denoted by *p*(*t_i_*), which has been measured for the time samples *t_i_*. For the sake of brevity, we will characterize *p*(*t_i_*) as *p_i_* in the following discussion. In addition, there is an m number of sets $p_{i}^{m}$ for each device *m*, which provides the power of that device at interval *i*. This power is usually measured with the help of a submeter installed at the device location. The occurrence of a change in aggregate power is a critical point in NILM, hereafter called an ‘event’ as denoted in literature [28], which can be described as *Δpi = p*_*i +* 1_ *− pi* and *Δ*$p_{i}^{m}$ = $p_{i+1}^{m}$ *−* $p_{i}^{m}$. Then mathematically, aggregate power can be expressed as [43]:

(5) $$p_{i}=\overset{\left|M\right|}{\underset{m=1}{\sum }}p_{i}^{m}+n_{i}$$

Here *n_i_* is the noise in the aggregate signal; this noise comprises measurement noise, unknown devices, and any baseload. For evaluating, $p_{i}^{m}$ within *p*(*t_i_*) set, i.e., the function given below must be minimized to achieve disaggregation. The nearer *f*(*r_i_*) is to zero; the greater is the evaluated $p_{i}^{m}$.

(6) $$f\left(r_{i}\right)=\|\Delta \;pi-\overset{\left|M\right|}{\underset{m=1}{\sum }}\Delta \;p_{i}^{m}{\|}_{2}^{2}$$

where $r_{i}=\left[\Delta p_{i}^{1},\Delta \;p_{i}^{2},\ldots ..,\Delta \;p_{i}^{m}\right]$ [41,52].

A graph is generated for employing GSP and spectral clustering to tackle this problem. The graph $G=\left(V,A\right)$ is generated, where each sample of *pi* is associated with the corresponding *vertex* v_i_ ϵ V and *A* is the adjacency matrix. For reference purposes, it is assumed that $p_{i}^{m}$ is available for *i* = 1,2,…,n < N for each device m ϵ M. Then the value of $p_{i}^{m}$ is used to set the thresholds for identifying the events of a particular device. The goal is to estimate $p_{i}^{m}$ for n < *i* ≤ N.

To disaggregate $p_{i}^{m}$ from *p*(*t_i_*), we first cluster the data based on their spectrum as presented by Stella et al., [53]. This clustering technique is modified for the data suiting NILM, i.e., power. Here each cluster in a moving window belongs to a specific device. Therefore, the cluster and thus the device profile is to be identified. Once a cluster and the device to which it belongs are determined, the power profile of each device m can be identified successfully. The two techniques proposed here and detailed below differ in methodology at the stage of cluster/device identification are feasible for NILM.

### 2.1. The Spectral Cluster-Mean Method

This method is designated as spectral cluster mean (SC-M) method, as the cluster’s mean has a significant role in cluster identification and error reduction. In the first step, adjacency matrix *A* is calculated according to Equation (1). According to the adjacency matrix, a linear similarity graph is defined with consecutive nodes connected and others unconnected with weights. In the following steps, the degree matrix and un-normalized Laplacian matrix are calculated in Equations (2) and (3), as elaborated in the introduction section. Next, *U* ϵ *N × k* is computed; it is the matrix containing *k* generalized eigenvectors *u*_1_*, u*_2_*, …, u_k_*, calculated from the generalized eigenvalue problem *Lu =* λ*u* as its columns. For each row of *U*, *y_i_* ϵ *k* is the corresponding vector. As the last classification step, *y_i_* is divided into clusters *C*_1_*, C*_2_*,…, C_k_*, using *k* means [47].

**Figure 4:**
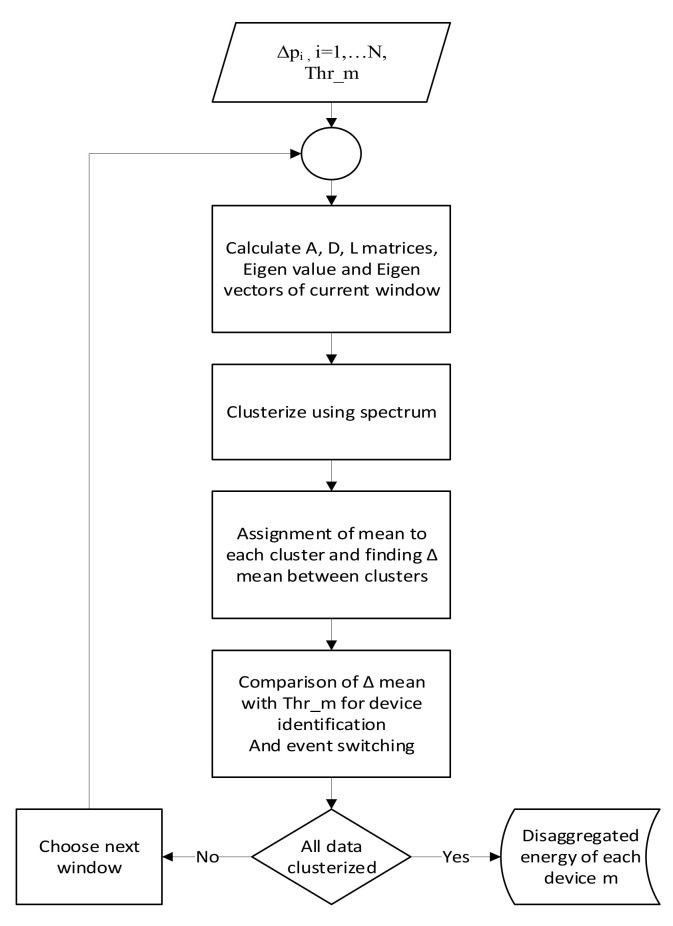
Flow chart of SC-M algorithm.

At this point in the calculations, a novel variation is introduced. The mean of each cluster is calculated and each of the cluster values is replaced by the cluster mean. The mean value reduces noise, avoids errors, and facilitates device recognition. To keep the mean and scatter of original data sufficiently close, the window size to be clustered is kept small. The authors kept the data size to 25 samples in each iteration window for this study. The data size of 25 samples was found heuristically. This dataset is a sliding window and keeps moving to encompass the upcoming samples. This moving window can be used on actual real-time signals. This approach will also help in a real-time application for NILM. According to the device’s specifications, a power threshold for each appliance *m* is assigned as *Thr_m_* ≥ 0. The threshold value is used in the evaluation and referencing stage. Whenever *ΔMean* of each consecutive cluster lies within the threshold range, it is assumed that a change in the state of that particular device has occurred. This technique is novel in its use for NILM. The current study is the first to use spectral clustering for electrical load disaggregation, with certain modifications introduced such as mean assignment and small window processing. After noting the *Δp_i_* of consecutive clusters and comparing them with the *Thr_m_* of each device, the cluster is designated to each device *m,* and its state change is estimated for each time *i*. The whole process is described in the block diagram below in Figure 4.

### 2.2. The Spectral Cluster Eigenvector Method

As in the second method, eigenvectors play a significant role in clustering and event identification; this method is designated as the spectral cluster eigenvector (SC-EV) method. The second method differs from the first method at the event detection stage. In the second method, the spectrum is utilized to detect an event and further recognize the device. Here, after clustering the input power stream using ‘*k*’ means [47], eigenvectors are further utilized for event detection and cluster/device identification.

Here, eigenvectors are added to form a resultant sum vector. A significant change in the resultant vector is observed whenever an event exists in the aggregate data. The authors have utilized this delta to initiate consecutive events. The magnitude of change (delta) in the sum vector is larger and unique in the case of each device switching. However, minor variations in the sum vector also exist because of unwanted variations in the main meter output. Therefore, a specific suitable value of threshold has to be utilized. A change above the threshold delta is then designated as an event.

Once an event is detected, its index is noted, and the delta of LS, which in the current case is an active power, is calculated at that particular index. In this way, the index of events from the aggregate power profile is automatically identified.

**Figure 5:**
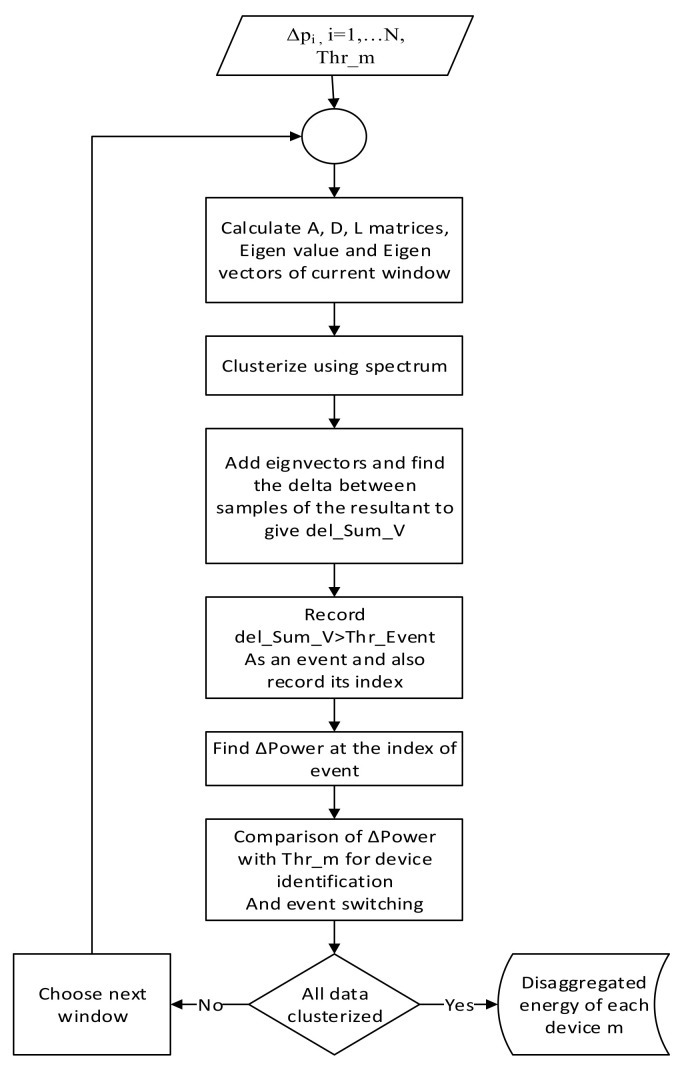
Flowchart of SC-EV algorithm.

Moreover, the respective change of magnitude of power is further used to identify the device or cluster. The change of magnitude of power *Δp_i_* is compared with device thresholds *Thr_m_* already declared according to the device specifications to recognize the device. Furthermore, it is noted that eigenvectors are the by-product of the spectral clustering algorithm, and no additional processing is required. This technique is thus the second novelty introduced by this work. Therefore, the second method has a substantial distinction from the first method. The process followed in the second method is described in the flowchart shown in Figure 5.

### 2.3. Performance Parameters

Although there are many parameters for evaluating machine learning algorithms and NILM techniques, the two most important parameters used for evaluation in the literature and this study are defined below [54].

(7) $$Accuracy=\frac{TP+TN}{TP+FN+TN+FP}$$

(8) $$F1=\frac{2\times precision\times recall}{precision+recall}=\frac{TP}{TP+0.5\times \left(FP+FN\right)}$$

where *TP*: true positive, *TN*: true negative, *FN*: false negative, and *FP*: false positive. Precision and recall have been defined in Appendix A.

## 3. Results

Two datasets are employed to test the performance of the above techniques. The first dataset is a clean (noiseless) and less complex (ideal) dataset containing the real power of the five devices for algorithm testing. These devices are two state devices. Sampling frequency is five samples per second. Later for real-world testing, the REFIT dataset [55] is used to test the accuracy of the developed algorithms. The REFIT dataset is the energy consumption profile of various devices of a UK-based housing community. This dataset has energy consumption data of 21 houses for approximately two years. The measurement frequency ranges from 4 to 10 samples. This sampling frequency lies in the low range of sampling and is sufficient for measuring steady-state load signature. The dataset is quite complex in real time as it contains unwanted transients, spikes, outliers, and abnormalities encountered in real-world measurements. Therefore, this dataset serves as a tough test for the NILM techniques. The dataset has a washing machine, PC, and TV as multistate devices. The authors have tested the presented NILM techniques with these two datasets and found valuable results, as presented in the following sections. As expected, the accuracies are very high in the ideal dataset and competitive with other unsupervised techniques in the case of the REFIT dataset.

### 3.1. Results of Spectral Cluster Mean Algorithm

The spectral cluster mean algorithm results, using the ideal dataset, are shown in Table 1. Before presenting the results on the actual REFIT dataset, it is pointed out that results for multistate devices like washing machines, PCs, and TVs are taken only for one major ‘ON’ state. That means they are assumed to be two states: ON/OFF devices to simplify the test. Next, the results of the same technique are presented on the real-world data REFIT in Table 2. Houses 1, 2, 3, 4, 5, 8, 9, 15, 17, 18, 19, and 21 are randomly selected from the 21 houses present in the dataset.

During the selection of houses, it is considered that solar energy is not present in the house, so the aggregate is not affected by it. Table 2 presents the mean performance parameters’ values of the devices (excluding outliers) for the houses mentioned above. The actual trend of performance is not affected by these outliers. The washing machine is not included in this table and is discussed in Section 4.

**Figure 6:**
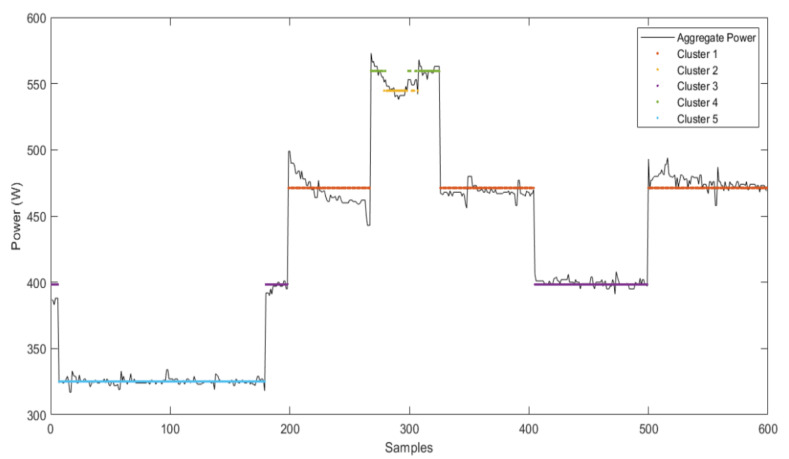
Example of REFIT data clustering using SC-M. According to the legend, different colors show separate cluster assignments of aggregate (black) and the mean of each cluster, which is further used for device/state identification.

It is evident in Figure 6 that by employing the spectral cluster mean algorithm, various changes of states are successfully clustered. The aggregate power shown in the graph is well-clustered using the algorithm, and each cluster’s mean and the cluster are shown in a different color. The mean value has an essential role in device identification.

### 3.2. Results of Spectral Cluster Eigenvector Algorithm

The spectral cluster eigenvector algorithm results on ideal data are presented in Table 3. By comparing Table 1 and Table 3, it is noted that the SC-EV algorithm outperforms the SC-M algorithm for the ideal dataset in some cases.

Next, the results of the SC-EV on the real-world data REFIT are presented in Table 4 and Table 5. The same number of houses are selected for comparison and analysis from the dataset. Table 4 and Table 5 also present the mean performance parameter values of the same devices of each house.

**Figure 7:**
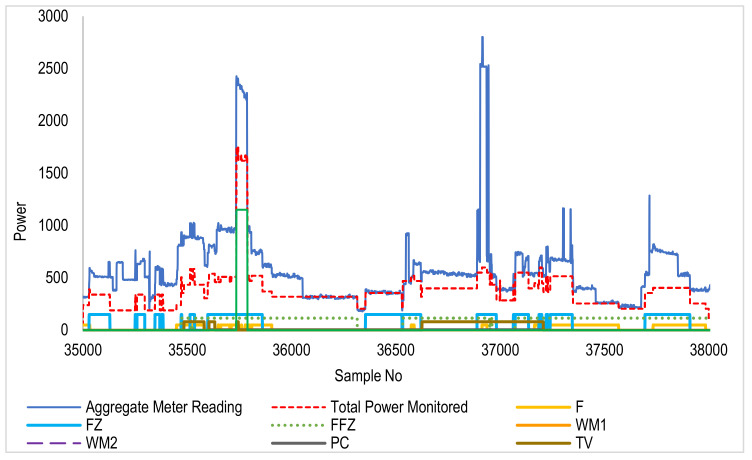
Aggregate and clustered REFIT data (samples: 35,000 to 38,000 window). As indicated in the legend, each device’s disaggregated profile is shown in a different color. The red plot is the sum of disaggregated devices’ energy. It is seen that the trend of sum (red) follows the aggregate (blue) at each instant showing the effectiveness of the technique.

**Figure 8:**
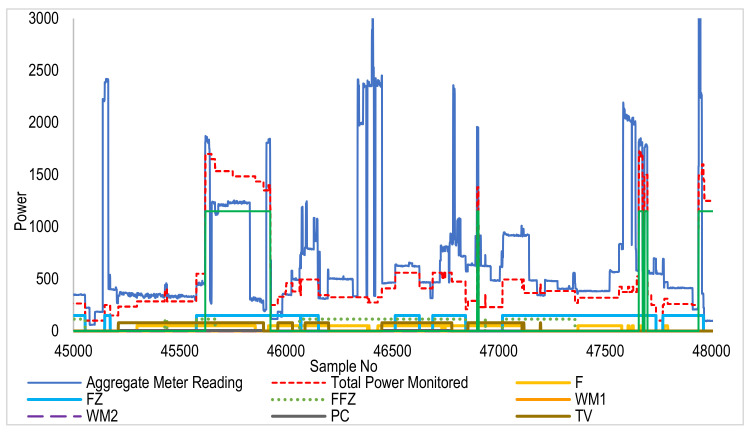
Aggregate and clustered REFIT data (samples: 45,000 to 48,000 window). As indicated in the legend, each device’s disaggregated profile is shown in a different color. The red plot is the sum of disaggregated devices’ energy. It is seen that the trend of sum (red) follows the aggregate (blue) at each instant showing the effectiveness of the technique.

Figure 7 and Figure 8 show the disaggregation of various devices from the aggregate signal. These data belong to House 4 of the REFIT dataset. A graph showing the sum of disaggregate devices is also shown in the dotted line, closely following the aggregate measured power. The difference between aggregate and sum also shows a variable baseload present throughout the dataset. In addition to that, some unknown loads and unwanted spikes are also present.

## 4. Discussion

In Table 1, the accuracy and f-measure of the first two devices lie in the range of 80 to 90% and that of Device 3 is somewhat lower. The main reason for lower performance in the case of SC-M lies in the misidentification of the cluster. In some cases, the SC-M assigns the mean to the cluster such that *ΔMean* does not lie within any of the *Thr_m_* ranges defined for cluster/device identification. This problem is mainly controlled by minimizing the window size to 25 samples; otherwise, the results can degrade. More research in this domain is needed on improving the cluster identification further, and more effective techniques can be used for future works. However, the SC-EV method handles this problem more efficiently.

Table 2 shows good accuracy in device identification of prominent loads, having high power consumption and being well-separated from other loads, e.g., dryer, microwave, kettle, toaster, pump, heater, and high-fidelity equipment. At the same time, the accuracy in identifying devices having less prominent state changes is lower. Similarly, appliances with overlapping power graphs, e.g., fridge/freezer, computer, and food mixer, are challenging to be identified. The main reason is that prominent devices get well-clustered initially and are easily segregated and identified later.

Table 3 presents the performance metrics of the SC-EV method for the ideal dataset. Most of the devices have an accuracy of 100%, while Device 2 has a misclassification and has an accuracy of 86%, which shows that SC-EV performs much better. Table 4 and Table 5 show the performance of SC-EV on the REFIT dataset. The difference between Table 4 and Table 5 lies in the threshold of the event. It is evident that this threshold has a vital role here, and an optimal balance needs to be achieved. Very narrow or broad threshold ranges deteriorate performance, and a delicate balance is needed in defining them.

The number of clusters within a data window also play a vital role in the performance of both techniques. An optimal balance needs to be achieved for this as well. Some significant and present events are not clustered if the number of clusters is kept too low. On the other hand, if a high number of clusters is selected, then some false events may be generated. These false events can decrease the accuracy of the disaggregation process. This phenomenon can be better visualized with the help of Figure 6, where an increase or decrease of the cluster affects the disaggregation accuracy of devices. Although in spectral clustering, the sizeable spectral gap between eigenvalues gives us a fair idea of the number of clusters present [47]. However, it has been observed that, in some cases, using this eigenvalue notion for determining the number of clusters is not practical because of the following reasons:

1. Too many events present in reality,
2. Some devices have meager power consumption,
3. Some devices have a nearly equal power rating or overlap each other,
4. Noise and variations magnitude in data is higher than the power rating of some devices.

The deciding factor is the number of devices present in the aggregate signal and the number of clusters used. It was determined heuristically that restricting the number of clusters to four for each 25-sample window provides the best results. A significant factor for this is that the number of events occurring within each 25-sample window also varies from two to four. However, the performance comparison of both approaches, i.e., the notion of a spectral gap between eigenvalues to determine the number of clusters with that of a user-defined number of clusters, has not yet been made and is left for future work.

It is also observed that the performance of compressor-based devices, i.e., fridge/freezers, remained low compared to other devices for both techniques. The main reason, observed here and reported before in the literature [43,54], is the cyclical pattern of these devices plus a complex decaying power profile during the ON state. This decaying power profile during the ON state changes the mean power from the initial transient, which sets during the ON state of the device. The other ON-state threshold is very different from the OFF-state threshold. As these techniques are based on thresholds, any threshold variation decreases accuracy. Furthermore, it is observed that fridge and freezer power profiles lie in close vicinity of each other, making it very difficult to recognize them accurately. Fridge/freezer power profiles are also similar to the power profiles of some other devices, e.g., computers and TVs, which further reduces the disaggregation performance of the developed algorithms for these devices.

The power of computers and TVs remains variable throughout their operational duration. Therefore, it becomes difficult to detect and disaggregate them, as is evident from Table 2 and Table 4. Therefore, it is concluded that these techniques need to be further refined to cater to devices that have a decaying/variable power profile.

Despite low detection performance for some devices, the developed algorithms provide excellent detection accuracy for many devices. Additionally, the algorithms developed during this study have the advantage of low complexity and fast processing time (discussed in Section 4.3) in achieving the results.

As stated in Section 3.1, results for multistate devices like washing machines, PCs, and TVs are taken only for one primary state, as tabulated in this study. The operational duration of some devices such as washing machines is minimal, i.e., once a week. So, according to the relation of accuracy, Equation (6), average accuracy turns out to be artificially very high. Thus, this accuracy may not reflect the actual performance and show unrealistically high accuracy. To cater to this, a smaller portion of data, having the significant operation of the washing machine, is selected, and its performance is evaluated. These statistics are presented in Table 6 and Table 7 with an asterisk mark (*) to differentiate them from average statistics.

### 4.1. Comparative Analysis of SC-M and SC-EV

**Figure 9:**
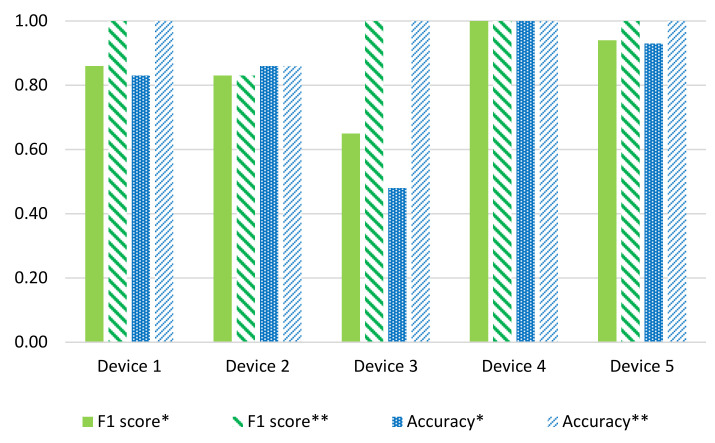
Performance (F1 score and accuracy) comparison of * SC-M and ** SC-EV for ideal data. The F1 score of both techniques is shown in green, while the accuracies of both techniques are in blue shades. Ideal data exhibits better performance than REFIT data because of low randomness and variation.

**Figure 10:**
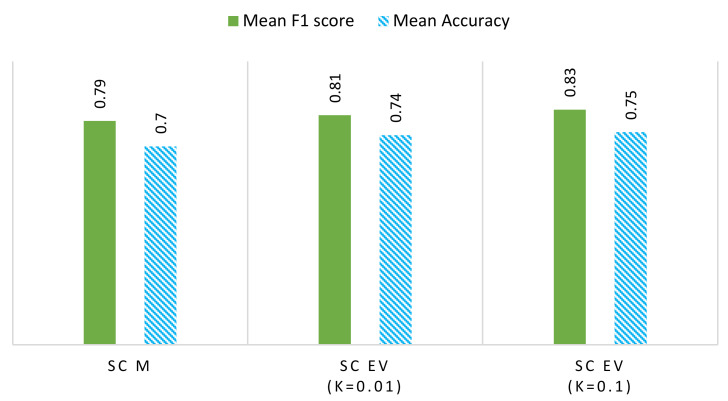
Performance (average F1 score and average accuracy) comparison of SC-M, SC-EV (*k* = 0.01), and SC-EV (*k* = 0.1) for REFIT data, *k* being the threshold for event detection. The figure shows that SC-EV outperforms SC-M in many cases.

Both methods are compared in terms of their f-measure score and accuracy. Figure 9 presents the comparison of these two methods for the ideal dataset. The SC-EV method outperforms the SC-M method generally, except in one case, i.e., Device 4, in which both perform equally. Figure 10 represents this comparison for the REFIT dataset. In this analysis, it is also evident that the SC-EV method is performing well. In addition, a comparison of different threshold ‘*k*’ values is presented for the SC-EV method. The value *k* has a significant role in the performance of the SC-EV method. Detailed reasoning is presented in the previous section and summarized here. SC-M has lower performance as it works on the cluster’s mean, while the SC-EV algorithm uses eigenvector magnitudes for device detection. Using eigenvector magnitudes improves device detection by enhancing the differences in the device signatures. Hence, the SC-EV algorithm performs better in most cases.

Figure 10 shows a comparison of mean accuracy and f-measure scores, illustrating that event threshold ‘*k*’ affects the detection accuracies. Setting *k* = 0.1 provides better performance as compared to *k* = 0.01. The effects of selecting the value of ‘*k*’ are highlighted in this study. However, a more detailed sensitivity analysis of the ‘*k*’ value is planned to be carried out in future work.

### 4.2. Comparison with State of the Art

**Figure 11:**
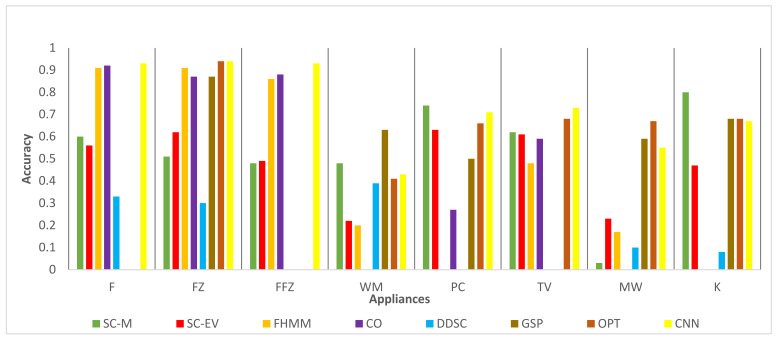
REFIT dataset House 4 comparison with results (accuracy).

**Figure 12:**
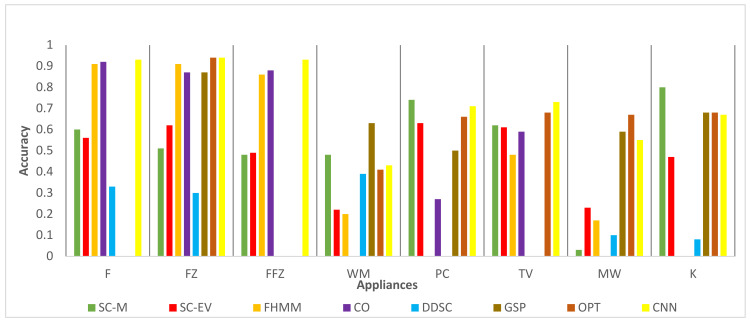
REFIT dataset House 8 comparison with results (accuracy).

Comparative performance analysis of the algorithms developed in this study with some of the latest results available in the literature is presented here. Recent research work by Zhao et al. [56] provides a detailed comparison of different NILM techniques’ disaggregation accuracies. The results (accuracies) provided by Zhao et al. have been compared with the current study results in Figure 11 and Figure 12 for the REFIT dataset. The benchmark studies are factorial hidden Markov model (FHMM), discriminative disaggregation sparse coding (DDSC), graph signal processing (GSP), unsupervised optimization (OPT), and convolutional neural network (CNN). Although optimization-based approaches, e.g., FHMM and OPT, have high accuracy for compressor-based normally on devices like F and FZ, they show reduced accuracy for other devices like PC, K, and WM. This is in line with NILM results reported previously [56,57]. On the other hand, proposed schemes SC-M and SC-EV exhibit consistent and robust performance across all devices generally. An exception of the MW exists here, which exhibits reduced accuracy as compared to other devices. However, the accuracy of MW detection remained high in other houses as shown in Table 4 and Table 5. The reason of low MW accuracy in these houses needs further investigation. In these figures WM results are given for short duration for the proposed schemes as discussed in previous section.

Furthermore, the presented results of both the SC-M and SC-EV methods are compared with another recent study regarding GSP on NILM [43]. Houses 2 and 17 of the REFIT dataset are used in the reference paper. The comparison is presented in Table 6 and Table 7. The algorithms proposed by this study provide better or competitive results of accuracy (Acc_m_) and f-measure (FM) metrics for most devices. However, considering the low computational complexity for an unsupervised technique, algorithms proposed in the current study may be more appropriate for real-time implementation in the NILM field.

**Table 6:** Comparison between proposed and state of the art methods in literature.

House		REFIT House 2							REFIT House 17						
		FFZ	DW	WM	TV	K	T	MW	WM	K	FFZ	TV	MW	Com	FZ
SC-M	FM	0.73	0.76	0.95 *	0.91	0.97	0.90	0.61	0.95 *	0.95	0.67	0.77	0.70	0.58	0.83
	Acc	0.61	0.62	0.92 *	0.84	0.95	0.82	0.44	0.91 *	0.90	0.63	0.64	0.54	0.46	0.76
SC-EV	FM	0.68	0.80	0.92 *	0.91	0.64	0.84	0.64	0.96 *	0.73	0.66	0.76	0.70	0.60	0.83
	Acc	0.57	0.67	0.85 *	0.84	0.48	0.72	0.47	0.94 *	0.57	0.63	0.62	0.54	0.48	0.76
UGSP [57]	FM	0.42	0.79	-	-	-	-	-	0.76	0.84	0.50	-	-	-	-
	Acc	0.77	0.42	-	-	-	-	-	0.53	0.79	0.66	-	-	-	-
SGSP [41]	FM	0.59	0.73	-	-	-	-	-	0.77	0.96	0.82	-	-	-	-
	Acc	0.8	0.67	-	-	-	-	-	0.61	0.80	0.70	-	-	-	-
DT [58]	FM	0.54	0.73	-	-	-	-	-	0.78	0.95	0.82	-	-	-	-
	Acc	0.73	0.61	-	-	-	-	-	0.52	0.77	0.67	-	-	-	-

**Table 7:** Comparison of mean f-measure between proposed and state-of-the-art for refit data.

House		House 2	House 17
**FM**	SC-M	0.86	0.82
	SC-EV	0.81	0.80
	P-UGSP	0.59	0.53
	P-SGSP	0.61	0.62
	P-DT	0.59	0.60

**Figure 13:**
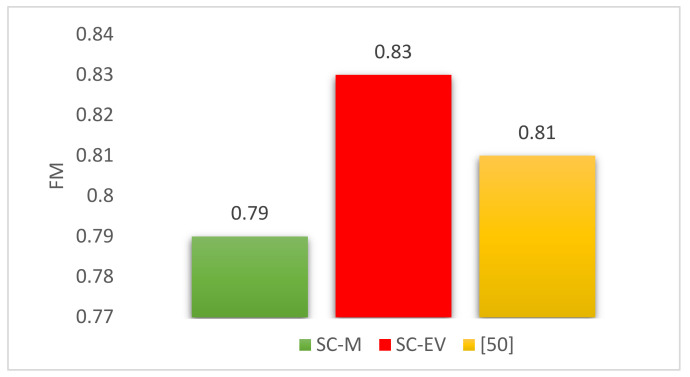
f-measure comparison of proposed and benchmark study [50].

A comparison of current approaches with two studies employing different approaches of spectral clustering technique may be of interest here, shown in Figure 13. In [50], the EMBED dataset was used to test three different clustering approaches. The non-iterative one-step approach has been chosen from the three approaches because the proposed approaches i.e., SC-M and SC-EV, are also non-iterative in nature. The mean score for the proposed, as well as all four datasets in the benchmark study from the said category is presented for comparison here. Although a comparison of REFIT performance with EMBED is debatable here because of the difference of complexity in these datasets. However, their comparison is interesting and relatable and provides further insight into the techniques.

### 4.3. Computation Time

Time elapsed during the processing of four days of data (50,578 samples) using the SC-M algorithm came out to be 23.89 s, or a per-sample computation time of 0.472 ms on an Intel Core i5-3427U, 1.8 GHz, 2.30 GHz, 8GB RAM, Windows 10 64-bit system with Matlab 2020a installed. Similarly, when processing four days of data (72,500 samples) using the SC-EV algorithm, the time elapsed is 43.44 s or a per-sample computation time of 0.599 ms. For comparison, a study [41] provides computational time data of two techniques: first, for a GSP-based technique, the study reports processing time for 20,000 samples to be 10 to 12 s or an average per-sample computation time of 0.55 ms. The system description for this study was an Intel Core i5-3470 CPU, 3.20 GHz processor running Windows 7 64-bit. The second study presents an HMM-based technique, with a processing time of 40–50 s for 20,000 samples, resulting in an average per-sample computation time of 2.25 ms. The preceding results show that algorithms developed in the current study are faster and more appropriate for real-time applications. The SC-M algorithm is a little faster than the semi-supervised GSP-based technique and much faster than the HMM-based technique, while accuracy and f-measure score are competitive and even better in many cases, keeping in mind that the proposed algorithms require no training data.

## 5. Conclusions

This paper demonstrates that NILM is a practical and economical solution for device-level energy monitoring. Although spectral clustering has been used to classify data, including images, its application in NILM has not been investigated. This paper proposes two novel unsupervised strategies: SC-M and SC-EV, which work on graph-based as well as non-graph-based data.

Despite their relatively poor performance for devices such as fridge-freezers, the proposed techniques show excellent detection accuracies for most devices. After successful testing on several datasets, results show a high f-measure of 0.79 and 0.83 together with an accuracy of 0.70 and 0.75 for SC-M and SC-EV, respectively. The results achieved by the proposed algorithms are found to be comparable or better in accuracy with respect to other recent studies. The improvement in accuracy is evident, especially when similar unsupervised GSP-based NILM techniques are considered.

Due to their higher accuracy, no training data requirement, shorter processing time, and less computational complexity, the proposed techniques can be applied to real-time and low-frequency smart meter data. These techniques can lead to better energy management and conservation in buildings. From a futuristic point of view, more avenues of research exist in improving these techniques, especially for f-measure score and accuracy. This paper also reveals several areas for further studies and possible improvements, including intelligent decision-making replacing the heuristic decisions in this work. Similarly, exploring the efficacy of these methods using different types of load signatures is recommended.

## Figures and Tables

**Table 1:** Performance of SC-M algorithm applied on ideal data.

	Device 1	Device 2	Device 3	Device 4	Device 5
f-measure score	0.86	0.83	0.65	1	0.94
Accuracy	0.83	0.86	0.48	1	0.93

**Table 2:** Performance of SC-M algorithm applied on the REFIT dataset.

	Fridge (F)	Freezer (FZ)	Fridge-Freezer (FFZ)	Washer Dryer (WD)	Dishwasher (DW)	Computer (PC)	Television (TV)	Microwave (MW)	Food-Mixer (FM)	Kettle (K)	Toaster (T)	Bread Maker (BM)	Heater (H)
f-measure score (Avg)	0.57	0.58	0.57	0.89	0.85	0.67	0.80	0.89	0.74	0.82	0.82	0.84	0.86
Accuracy (Avg)	0.46	0.48	0.59	0.82	0.63	0.53	0.67	0.82	0.60	0.79	0.76	0.74	0.78

**Table 3:** Performance of SC-EV algorithm on ideal data.

	Device 1	Device 2	Device 3	Device 4	Device 5
f-measure score	1	0.83	1	1	1
Accuracy	1	0.86	1	1	1

**Table 4:** Results of SC-EV algorithm applied on REFIT data with *k* = 0.01.

	F	FZ	FFZ	WD	DW	PC	TV	MW	FM	K	T	BM
f-measure score (Avg)	0.50	0.64	0.60	0.86	0.90	0.58	0.76	0.94	0.76	0.87	0.86	0.82
Accuracy (Avg)	0.40	0.57	0.60	0.78	0.83	0.45	0.75	0.90	0.61	0.74	0.80	0.69

**Table 5:** Results of SC-EV algorithm applied on REFIT data with *k* = 0.1.

	F	FZ	FFZ	WD	DW	PC	TV	MW	FM	K	T	BM
f-measure score (Avg)	0.60	0.72	0.60	0.86	0.85	0.67	0.81	0.93	0.76	0.92	0.86	0.82
Accuracy (Avg)	0.40	0.63	0.61	0.78	0.76	0.59	0.79	0.88	0.61	0.73	0.76	0.69

## Data Availability

Publicly available dataset REFIT used in this study is available at the following link: https://www.refitsmarthomes.org/datasets/, accessed on 20 April 2022.

## Appendix A

Precision and recall in Equation (7) is defined as follows:

(A1) $$\mathrm{Precision}=\frac{\mathrm{TP}}{\mathrm{TP}+\mathrm{FP}}$$

(A2) $$\mathrm{Recall}=\frac{\mathrm{TP}}{\mathrm{TP}+\mathrm{FN}}$$

where *TP*: true positive, *TN*: true negative, *FN*: false negative, and *FP*: false positive.
